# Increased retinal drusen in IgA glomerulonephritis are further evidence for complement activation in disease pathogenesis

**DOI:** 10.1038/s41598-022-21386-y

**Published:** 2022-10-31

**Authors:** P. Harraka, Tony Wightman, Sarah Akom, Kieran Sandhu, Deb Colville, Andrew Catran, David Langsford, Timothy Pianta, David Barit, Frank Ierino, Alison Skene, Heather Mack, Judy Savige

**Affiliations:** 1 Department of Medicine, Northern Health, The University of Melbourne, Parkville, VIC 3050 Australia; 2 Department of Medicine, Melbourne Health, The University of Melbourne, Parkville, VIC 3050 Australia; 3 Department of Nephrology, Austin Health, The University of Melbourne, Parkville, VIC 3050 Australia; 4 Department of Pathology, Austin Health, The University of Melbourne, Parkville, VIC 3050 Australia; 5 The University of Melbourne Department of Ophthalmology, Royal Victorian Eye and Ear Hospital, East Melbourne, VIC 3002 Australia

**Keywords:** Biomarkers, Diseases, Medical research, Nephrology, Pathogenesis

## Abstract

Drusen are retinal deposits comprising cell debris, immune material and complement that are characteristic of macular degeneration but also found in glomerulonephritis. This was a pilot cross-sectional study to determine how often drusen occurred in IgA glomerulonephritis and their clinical significance. Study participants underwent non-mydriatic retinal photography, and their deidentified retinal images were examined for drusen by two trained graders, who compared central drusen counts, counts ≥ 10 and drusen size with those of matched controls. The cohort comprised 122 individuals with IgA glomerulonephritis including 89 males (73%), 49 individuals (40%) of East Asian or Southern European ancestry, with an overall median age of 54 years (34–64), and median disease duration of 9 years (4–17). Thirty-nine (33%) had an eGFR < 60 ml/min/1.73 m^2^ and 72 had previously reached kidney failure (61%). Overall mean drusen counts were higher in IgA glomerulonephritis (9 ± 27) than controls (2 ± 7, *p* < 0.001). Central counts ≥ 10 were also more common (OR = 3.31 (1.42–7.73, *p* = 0.006), and were associated with longer disease duration (*p* = 0.03) but not kidney failure (*p* = 0.31). Larger drusen were associated with more mesangial IgA staining (*p* = 0.004). Increased drusen counts were also present in IgA glomerulonephritis secondary to Crohn’s disease but not with Henoch-Schonlein purpura. The finding of retinal drusen in IgA glomerulonephritis is consistent with complement activation and represents a model for better understanding glomerular immune deposition and a supporting argument for treatment with anti-complement therapies.

## Introduction

IgA nephropathy is the commonest form of glomerulonephritis worldwide^1^, affecting men more often than women, and especially people of East Asian or Southern European ancestry^2^. IgA disease is characterised by mesangial deposits of IgA, IgG, and C3. Typical clinical features include episodes of macroscopic hematuria, persistent microscopic hematuria, proteinuria, hypertension, and, kidney failure in 20% after 20 years^3^.

The pathogenesis of IgA nephropathy is incompletely understood, but onset and relapse are often associated with respiratory and gastrointestinal tract infections. IgA itself represents the first line of defence against mucosally-invasive bacteria, and individuals with IgA nephropathy have increased levels of the galactose-deficient form of IgA1^4^, as well as glycan-specific autoantibodies^5^. The mesangial deposition of IgA1-IgG complexes damages the renal podocytes, resulting in glomerular and tubular interstitial damage. This reaction is amplified by IgA- induced activation of the alternative^6^ and lectin^7^ complement pathways, through activation by mannose binding lectin (MBL)-associated serine proteases (MASP2). Further evidence for complement involvement in IgA nephropathy includes the observations that mesangial C3 deposits and low circulating C3 levels are both associated with a worse outcome^8^.

Genetic factors are also important in the pathogenesis of IgA nephropathy. Families are affected in at least 15% of cases^9^ where inheritance is autosomal dominant^10^. GWAS have identified associations with more than 100 genes but each appears to have a small effect and together they account for less than 10% of the total genetic risk^11^. These include associations with complement regulatory genes at as well as other loci at 2q36 (*COL4A3/COL4A4)*^12^; 6p21 (*HLA-DQ-DR, TAP1)*; 8p23 (*DEFA*); 17p13 (*TNFSF13*); and 22q12 (*HORMAD2*)^11,13^. Mutant genes have also been identified in rare families and these often affect complement pathway proteins^14^, including *CFHR1-3* at 1q32^13^, and *C3, C1q, C2, C4, C9,* and *CFB*^15–19^.

Recent reports have described retinal drusen in IgA nephropathy^20–22^. Drusen are yellow-white deposits visible on ophthalmoscopy and in imaging, that also occur in other forms of glomerulonephritis such as SLE^23^ dense deposit disease^24,25^, post-streptococcal and membranous glomerulonephritis^26^. Occasional drusen are normal in middle-age but increased numbers of large, soft, mainly foveal deposits are usually typical of age-related macular degeneration. Drusen composition in macular degeneration resembles that of the immune complexes in glomerulonephritis with lipid, immunoglobulins, complement, CRP, and vitronectin^26,27^.

In contrast to immune complex formation in glomerulonephritis, drusen pathogenesis and progression in macular degeneration are reasonably well-understood^28^. Drusen risk factors include genetics, age, smoking, hypertension, renal failure^29^ and systemic inflammation^30,31^. Thirty-four genes have been implicated^32^ encoding proteins for complement activation, lipid metabolism, extracellular matrix integrity, energy production, apoptosis and angiogenesis^33–35^. These suggest that membrane lipid (from apoptotic cells in the retina) is altered by reactive oxygen species, which activates complement, initiating an immune reaction and recruiting inflammatory cells. Defective extracellular matrix enables the drusen to enlarge, but over time the drusen are resorbed, resulting in retinal atrophy.

This study investigated individuals with IgA glomerulonephritis for drusen and their clinical significance.

## Patients and methods

### Study design

This was a pilot observational cross-sectional study of individuals with IgA glomerulonephritis recruited consecutively from a general nephrology or transplant clinic at two centres over a four year period. Recruitment, data capture and retinal imaging were coordinated in a single episode, and retinal photographs were examined for drusen. Results were compared with those from matched controls who attended a general medical clinic.

The primary outcome was to determine if drusen were more common in individuals with IgA glomerulonephritis than in other hospital patients, and the secondary outcomes were to determine if drusen were associated with longer disease duration or a risk of end-stage kidney failure. There were no changes to the study design after its commencement and no interim analyses.

This project was approved by the Austin Health and Northern Health Research Ethics Committees, according to the principles of the Declaration of Helsinki, and each participant provided written informed consent.

### Participants

Consecutive individuals with IgA glomerulonephritis diagnosed on conventional histopathologic criteria by a renal pathologist or where there was a strong index of clinical suspicion and who were attending a routine clinic review for management of their kidney disease were invited to participate. All had been managed according to current protocols.

Control participants were age- and sex-matched hospital patients without systemic inflammatory or kidney disease who were recruited contemporaneously from general medical clinics.

The only exclusion criteria were retinal images that were bilaterally ungradable for drusen.

### Measurements

#### Clinical features

Participants provided a brief medical history (self-reported ancestry, age, sex, kidney transplant status) and drusen risk factors (smoking, hypertension, diabetes), and their charts were reviewed for current eGFR measurements and renal biopsy results. Associated inflammatory bowel disease, chronic liver disease, Henoch-Schonlein purpura and familial kidney disease were noted.

#### Retinal imaging and grading for drusen

Participants then underwent retinal imaging centred on the macula and disc of both optic fundi using a non-mydriatic retinal camera (CR-45, Canon, Japan). Deidentified images were examined by two trained graders independently, and drusen counted according to a grid overlay corresponding to the Wisconsin Age-Related Maculopathy Grading System^36^. This method was highly reproducible with an intraclass correlation of 0.94.

Drusen numbers were recorded from the retina with the higher count. Central drusen were counted in four separate quadrants of the fovea and parafovea^23^. Counts ≥ 10 centrally were considered abnormal^37^. Peripheral drusen were at least two disc diameters from the fovea.

Drusen size was assessed by convention by comparison with the span of the largest arteriole and venule where they crossed the disc margin (63 µm and 125 µm) as: small (≤ 63 µm), medium (63–125 µm) or large (> 125 µm)^23^. Retinal atrophy and pigmentation were noted by a retinal expert ophthalmologist. Some individuals underwent further tests, including optical coherence tomography (OCT, Zeiss, Germany) and autofluorescence.

### Statistical analysis

This was a pilot study to determine if drusen occurred more often in IgA nephropathy than controls, and if so, to generate hypotheses of their clinical significance. The statistics were not corrected for multiple analyses in order to ascertain all possible associations.

Categorical variables were compared using Fisher’s exact test. Continuous variables were compared with the student’s t-test, one-way ANNOVA or the Mann-Whitney test if non-normally distributed. Analyses were performed using SPSS v25 (IBM, US). A *p*-value of less than 0.05 was considered significant, and a *p*-value between 0.05 and 0.10 was considered a trend.

## Results

### Demographic and clinical features

One hundred and twenty-two unrelated individuals with IgA glomerulonephritis, including 89 men (73%) and 33 women (27%), with a median age of 54 years (IQR 39–64) were studied (Table 1). Twelve of these also had Henoch-Schonlein purpura^38^, four had inflammatory bowel disease, two had chronic liver disease and 3 had a family history of haematuria or renal impairment.

**Table 1:** Demographic and clinical characteristics of individuals with IgA glomerulonephritis or controls.

Clinical characteristics	IgA nephropathy (n = 122)	Controls (n = 122)	OR (95% CI), *p*-value
Age in years, median (IQR)	54 (39–64)	53 (39–61)	*p* = 0.59
**Sex, n (%)**			
Male	89 (73%)	89 (73%)	1.00 (0.57–1.76), 1.00
Female	33 (27%)	33 (27%)	
**Ethnicity, n (%)**			
East Asian plus Southern European	49 (40%)	36 (30%)	0.62 (0.37–1.06), 0.11
Northern European	73 (60%)	86 (70%)	
**Co-morbidities, n (%)**			
Hypertension	105 (86%)	43 (35%)	**11.3 (6.03–21.4), < 0.001**
Diabetes	22 (18%)	23 (19%)	0.95 (0.50–1.81), 1.00
Smoking history	30/108 (28%)	50/120 (42%)	**0.54 (0.31–0.94), 0.04**
Disease duration in years, median (IQR) (n = 103)	9 (4–17)	NA	NA
**Kidney function (n = 119)**			
eGFR (ml/min/1.73m^2^), median (IQR) (n = 58) pre–transplant	40 (20–73)	90	NA
Normal eGFR	8 (7%)		
CKD 2–4, n (%)	39 (33%)		
**Kidney failure, n (%)**	72 (61%)		
Dialysis, n (%)	6 (5%)		
Transplant, n (%)	61 (51%)		
Age at transplant, mean ± SD	46 ± 13		
**Renal biopsies (n = 30) (%)**			
Mesangial IgA			
Strong (3 +)	7 (23%)	NA	NA
Moderate (2 +)	18 (60%)		
Weak (1 +)	5 (17%)		
Mesangial C3 or C1q	23 (77%)		
No complement staining	7 (23%)		

*CKD* Chronic kidney disease, *eGFR* Estimated glomerular filtration rate mL/min/1.73m^2^, *IQR* Interquartile range, *OR* Odds ratio, *CI* Confidence interval, *NA* Not applicable.

Significant values are in bold.

Twenty-six (21%) were of East Asian (Chinese) and 23 (19%) of Southern European ancestry. One hundred and five had hypertension (86%), 22 (18%) had diabetes and 30 (28%) were current or former smokers. Their median disease duration was 9 years (IQR 4–17). Eight had a normal eGFR (7%), 39 (33%) had renal impairment (eGFR < 90 ml/min/1.73 m^2^), and 72 (61%) had reached end-stage kidney failure and were on dialysis, awaiting dialysis, had refused treatment (n = 11, 9%), or had already undergone kidney transplantation (n = 61, 51%).

Renal biopsies were reviewed in 30 cases. Mesangial IgA staining was 3 + (n = 7), 2 + (n = 18) or 1 + (n = 5), and complement staining (C3 or C1q) was positive in 23 (77%) biopsies.

Individuals with IgA glomerulonephritis were age- and sex-matched with hospital controls and were more likely to have hypertension (OR 11.3 (6.03–21.4), *p* < 0.001, and renal impairment, but were less likely to be smokers (OR = 0.54 (0.31–0.94), *p* = 0.04) (Table 1). They were not different in ancestry or likelihood of diabetes.

### Drusen

All individuals with IgA glomerulonephritis and all controls had at least one gradable retinal image. However drusen were counted using only one image for 22 (18%) of those with IgA nephropathy and 27 controls (22%).

**Figure 1:**
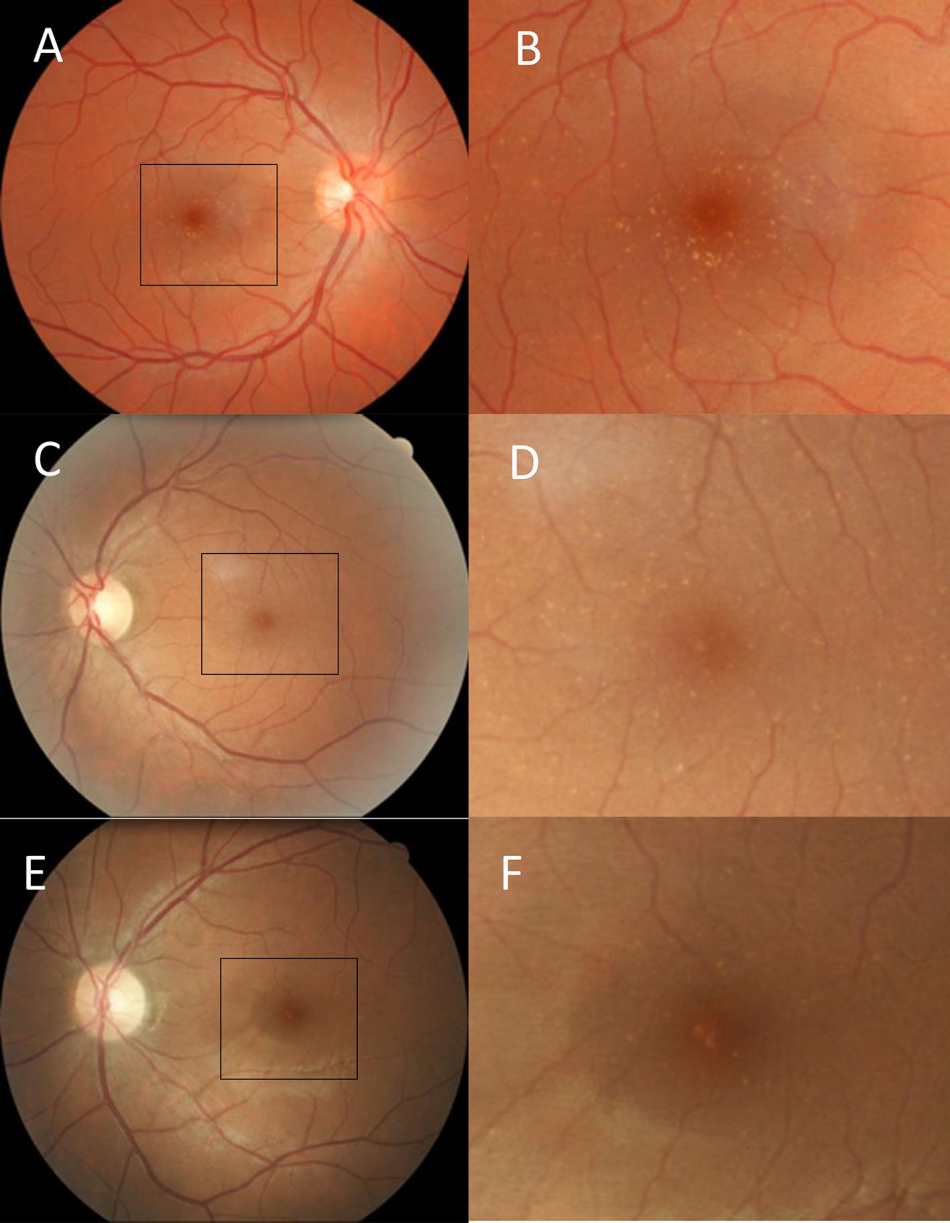
Retinal views of the central retina in three individuals with IgA glomerulonephritis (**A**,**C**,**E**) with enlarged views of the central retina demonstrating drusen (**B**,**D**,**F**).

Mean central drusen counts were 9 ± 27 in individuals with IgA glomerulonephritis and 2 ± 7 in the controls (*p* < 0.001) (Table 2) (Fig. 1). Twenty-three of those with IgA glomerulonephritis (19%) but only 8 (7%) controls had drusen counts ≥ 10 (OR 3.31 (1.42–7.73), *p* = 0.006)**.**

**Table 2:** Retinal abnormalities of individuals with IgA glomerulonephritis or controls.

Retinal abnormality	IgA (n = 122)	Control (n = 122)	OR (95% CI), *p*-value
Central drusen, Mean ± SD	9 ± 27	2 ± 7	***p* < 0.001**
≥ 10 drusen in worse eye Central, n (%)	23 (19%)	8 (7%)	**3.31 (1.42–7.73), 0.006**
Drusen in 4 macular quadrants, n (%)	25 (20%)	12 (10%)	**2.36 (1.13–4.95), 0.03**
**Drusen size, n (%)**			
Any small drusen	96 (79%)	72 (59%)	2.56 (1.46–4.51), 0.001
Any medium-large drusen	13 (11%)	17 (14%)	0.74 (0.34–1.59), *p* = 0.56
Pigmentation, n (%)	5 (4%)	1 (1%)	5.22 (0.60–45.3), 0.12
Atrophy, n (%)	4 (3%)	5 (4%)	0.80 (0.21–3.05), 1.00

*Central* Fovea and parafovea, *Peripheral* perifovea and non-macular, *SD* Standard deviation.

Significant values are in bold.

Any small drusen were found in 96 (79%) individuals with IgA glomerulonephritis and 72 (59%) controls (OR 2.56 (1.46–4.51), *p* = 0.001). There was no difference in the likelihood of medium-large drusen which were present in 13 individuals with IgA glomerulonephritis (11%) and 17 controls (14%) (OR 0.74 (0.34–1.59), *p* = 0.56).

Drusen affected all four of the central quadrants in 14 individuals with IgA glomerulonephritis (11%) and 7 controls (6%) (OR 2.13 (0.83–5.48), *p* = 0.17).

Retinal atrophy associated with drusen was present in 4 individuals with IgA glomerulonephritis (3%) and 5 (4%) controls (*p* = 1.00), and pigmentation was present in 5 with IgA nephropathy (4%) and one control (1%) (*p* = 0.12). None of the individuals with atrophy or pigmentation had more than 10 drusen in the central retina.

**Figure 2:**
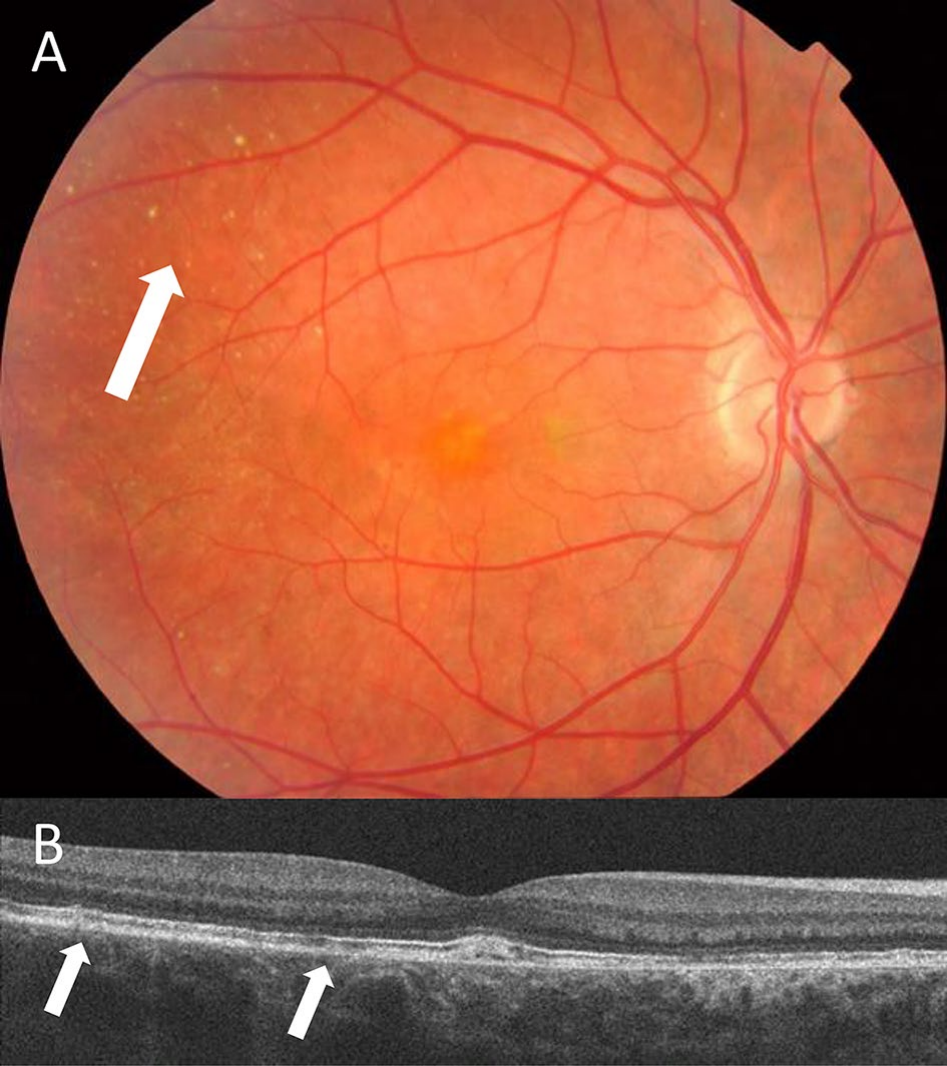
Retinal view of the central retina in an individual with IgA glomerulonephritis and ulcerative colitis demonstrating drusen (**A**) that are visible between the retinal pigment epithelium and Bruch’s membrane on optical coherence tomography (OCT, **B**).

Four individuals with IgA glomerulonephritis and drusen underwent further studies. All had normal visual acuity and visual fields. One who also had Crohn’s disease had normal Amsler grid testing (for macular degeneration). On OCT her drusen were located between the retinal pigment epithelium and Bruch’s membrane (Fig. 2). Another had abnormalities at both the inner limiting membrane and retinal pigment epithelium, but in the other two, the drusen were too small to be visualised by OCT.

### Clinical associations

Drusen counts in IgA glomerulonephritis were not increased with older age (0.12), sex (*p* = 0.71), ancestry (*p* = 0.91), hypertension (*p* = 0.14), diabetes (*p* = 0.34) or smoking history (*p* = 0.22) (Table 3). There was however a trend to an association with longer disease duration (*p* = 0.09).

**Table 3:** Demographic and clinical associations of drusen in individuals with IgA glomerulonephritis.

Clinical features	Central drusen, mean ± SD	*p*-value	≥ 10 drusen centrally, n (%)	OR (95%CI), *p*-value	Medium or large drusen, n (%)	OR (95%CI), *p*-value
**Age in years**						
< 40 (n = 31)	7 ± 25	0.12	3 (10%)	0.38 (0.10–1.38), *p* = 0.19	3 (10%)	0.86 (0.22–3.38), *p* = 1.00
≥ 40 (n = 91)	10 ± 28		20 (22%)		10 (11%)	
**Sex**						
Male (n = 89)	10 ± 27	0.71	18 (20%)	1.42 (0.48–4.19), *p* = 0.61	11 (12%)	2.19 (0.46–10.43), *p* = 0.51
Female (n = 33)	9 ± 30		5 (15%)		2 (6%)	
**Ethnicity**						
East Asian plus Southern European (n = 49)	8 ± 21	0.91	9 (18%)	0.95 (0.37–2.40), *p* = 1.00	6 (12%)	1.32 (0.41–4.18), *p* = 0.77
Northern European (n = 73)	11 ± 31		14 (19%)		7 (10%)	
**Co-morbidities**						
Hypertension (n = 105)	8 ± 24	0.14	17 (16%)	**0.35 (0.12–1.09), *p* = 0.09**	12 (11%)	2.06 (0.25–16.99), *p* = 0.69
No hypertension (n = 17)	20 ± 43		6 (35%)		1 (6%)	
Diabetes (n = 22)	11 ± 29	0.34	5 (23%)	1.33 (0.44–4.11), *p* = 0.56	4 (18%)	2.25 (0.62–8.10), *p* = 0.25
No diabetes (n = 100)	9 ± 27		18 (18%)		9 (9%)	
Smoking history (n = 30)	10 ± 32	0.22	6 (20%)	1.38 (0.46–4.07), *p* = 0.57	3 (10%)	0.97 (0.24–3.94), *p* = 1.00
No smoking (n = 78)	6 ± 18		12 (15%)		8 (10%)	
**Disease duration in years**						
≥ 10 (n = 53)	15 ± 37	**0.09**	16 (30%)	**3.32 (1.18–9.32), *p* = 0.03**	7 (13%),	1.43 (0.42–4.83), *p* = 0.76
< 10 (n = 52)	4 ± 10		6 (12%)		5 (10%)	
**Renal outcomes**						
Normal eGFR (n = 8)	1 ± 2	0.56	0 (0%)	*p* = 0.31	1 (13%)	P = 0.87
Impaired eGFR (n = 39)	12 ± 34		7 (18%)		5 (13%)	
Kidney failure (n = 72)	9 ± 25		16 (22%)		7 (10%)	
**Age at kidney failure in years**						
≥ 40 (n = 41)	12 ± 32	0.55	11 (28%)	1.93 (0.54–6.88), 0.38	5 (13%)	1.60 (0.29–8.93), 0.70
< 40 (n = 25)	4 ± 8		4 (17%)		2 (8%)	
**Mesangial deposits (n = 30)**						
IgA						
1 + (n = 5)	34 ± 73	0.97	1 (20%)	*p* = 0.76	0 (0%)	***p* = 0.004**
2 + (n = 18)	10 ± 15		5 (28%)		1 (6%)	
3 + (n = 7)	21 ± 51		1 (14%)		4 (57%)	
**C3/C1q**						
Present (n = 23)	20 ± 44	0.36	6 (26%)	2.12 (0.21–21.4), *p* = 1.00	3 (13%)	0.38 (0.05–2.88), *p* = 0.57
Absent (n = 7)	4 ± 7		1 (14%)		2 (29%)	

*eGFR* Estimated glomerular filtration rate mL/min/1.73m^2^, *SD* Standard deviation, *OR* Odds ratio, *CI* Confidence interval.

Significant values are in bold.

The mean drusen count was 1 ± 2 with normal kidney function (n = 8), 12 ± 34 with kidney impairment (n = 39) and 9 ± 25 after kidney failure had been reached (n = 72) but these differences were not significant (*p* = 0.56).

The mean drusen count was not associated with stronger mesangial IgA (*p* = 0.97) or complement staining (*p* = 0.36).

Drusen counts ≥ 10 at the fovea in IgA disease were not increased with older age (*p* = 0.19), sex (*p* = 0.61), ancestry (*p* = 1.00), diabetes (*p* = 0.56), or smoking history (*p* = 0.57) but surprisingly were less common with diagnosed hypertension (*p* = 0.09) (Table 3). However drusen ≥ 10 were more likely with longer disease duration (*p* = 0.03).

Drusen counts ≥ 10 were not present with in individuals with normal kidney function (n = 8), but were found in 7 of the 39 (18%) with renal impairment, and 16 of the 72 (22%) who had reached kidney failure (*p* = 0.31), many of whom had a kidney transplant.

Medium-sized and large drusen were not associated with any of the demographic risk factors, drusen risk factors, renal impairment or disease duration (Table 3). However larger drusen were associated with stronger mesangial IgA staining (*p* = 0.004).

### Other disease associations

Drusen were found in one person with IgA glomerulonephritis secondary to Crohn’s disease, two with a family history of IgA nephropathy, and none with chronic liver disease.

Twelve of the cohort also had Henoch-Schonlein purpura, where 11 were men (92%) and the overall median age was 39 years (IQR 32–57). Three had normal kidney function, 3 had impaired kidney function and six had reached kidney failure and received a kidney transplant. They were not different from the others with IgA nephropathy in terms of hypertension, diabetes or smoking history (*p* = 0.64, *p* = 1.00 and *p* = 0.66). Their mean total drusen count was 6 ± 6 which was also not different from IgA glomerulonephritis with 18 ± 51 (*p* = 0.42, diff = 12, 95%CI − 17.24–41.24). However none had central drusen ≥ 10 compared with 23 in the other 110 with IgA glomerulonephritis (21%) (*p* = 0.12, OR 0.15 (0.01–2.61). One had pigmentation and atrophy (8%).

Only one of the three individuals with recurrent transplant IgA glomerulonephritis had retinal drusen ≥ 10.

### Drusen and disease course

Drusen were noted first at presentation in some individuals with IgA glomerulonephritis. Eleven underwent retinal imaging a second time after a median of 3 years (1–9 years). Over this time, 5 (45%) had an increase in the number of drusen, 3 (27%) had a decrease and 3 (37%) had no change. Overall their mean total drusen count increased from 63 to 133.

The average central drusen count in people with a kidney transplant was 10 ± 27 (n = 61) compared with 21 ± 55 prior to transplantation (n = 58) (*p* = 0.67).

## Discussion

Retinal drusen are more common in IgA glomerulonephritis than in other age and sex-matched hospital patients. About 20% of individuals with IgA nephropathy have at least 10 drusen in one eye. Drusen counts and drusen ≥ 10 were increased with longer disease duration, and drusen size correlated with stronger mesangial IgA staining. While there was no correlation with renal impairment, the mean drusen count in IgA nephropathy was 1 ± 2 when kidney function was normal, and 9 ± 25 with kidney failure. Drusen were also present in IgA glomerulonephritis secondary to Crohn’s disease but not Henoch-Schonlein purpura. The finding of increased drusen counts in IgA nephropathy secondary to Crohn’s disease and the absence of drusen in Henoch-Schonlein purpura were consistent with persistent activity in Crohn’s disease and disease resolution in Henoch-Schonlein purpura.

This was an exploratory study of drusen in IgA glomerulonephritis that examined a moderately-sized cohort from two centres since only isolated case reports had been reported previously^20–22^. Drusen in IgA glomerulonephritis were located most often in the temporal central retina where the blood flow is maximal and debris collects, but were also found in clusters in the peripheral retina. Most drusen in IgA glomerulonephritis were smaller (< 63 um) than those found in macular degeneration and complications such as atrophy and pigmentation that occur with drusen resorption were uncommon^22^. If retinal imaging is insensitive for demonstrating drusen^23^ then drusen may be even more common in IgA glomerulonephritis, and our findings relate to the larger drusen that are visible on retinal imaging.

Drusen were not increased in people of East Asian or Southern European ancestry^39^ and were also not associated with the typical risk factors, of hypertension, smoking or kidney failure^29^. Interestingly drusen were also not associated with stronger mesangial complement staining.

Overall the drusen count in IgA glomerulonephritis appeared to increase on repeat retinal imaging. The cohort was too small to correlate drusen counts with worsening kidney function but our observations suggest that drusen accumulate with ongoing disease activity.

Retinal drusen also occur in other forms of glomerulonephritis including SLE, dense deposit disease, membranous and post-streptococcal glomerulonephritis^23–26^. Drusen occur in dense deposit disease, where kidney failure is typical, and severe visual loss develops^24,25^. The drusen in dense deposit disease may be associated with monogenic disease due to mutations in *CFH, C3* or *CFB*^25^*.* Drusen occur in 40% of individuals with SLE, in the same temporal retinal location as for IgA nephropathy^23^. Drusen in SLE are independent of kidney disease but drusen counts are greater, and drusen are larger and more widespread with lupus nephritis^23^. The reason for the difference in the likelihood of increased counts and significance of drusen in IgA nephropathy and SLE is not clear.

The composition of the subepithelial and subendothelial immune complexes found in membranous and post-streptococcal glomerulonephritis appears to resemble drusen composition in macular degeneration^26,27^. This includes apoptotic cell debris, membrane lipoproteins, immune globulins, complement, extracellular matrix, and CRP which suggest common pathogenetic features but there are also differences. In IgA nephropathy the glomerular immune deposits are mesangial rather than subepithelial or subendothelial, and drusen are much larger (≥ 63 µm) than the glomerular immune deposits (about 4 µm).

The genetics of drusen in macular degeneration are well understood. The two genes that account for more than half the risk are complement factor H (*CFH*)^40,41^, and *ARMS2/HTRA1*^42^ (age-related macular degeneration gene/high temperature requirement A-1)^32^. CFH is an inhibitory regulator of the alternative complement pathway, that reduces complement inactivation and increases membrane attack complex activity and subsequent damage to the retinal pigment epithelium. The commonest *CFH* variant, Y402H, is associated with increased drusen counts in macular degeneration. *HTRA1* encodes a serine protease that degrades extracellular matrix enabling drusen to enlarge.

IgA nephropathy and the drusen in macular degeneration share genetic risk alleles in complement pathway genes *CFH* and *CFHR1-5*^40,41^*.* Further major complement risk alleles in macular degeneration include *C2-CFB, CFI, C3* and *TIMP3*^28,33^*.* Although there are isolated reports of complement pathway risk alleles in IgA glomerulonephritis, the only major ones are *ITGAM* and *ITGAX* that code for integrins that facilitate complement-dependent phagocyte activation and immune complex clearance. Interestingly both IgA glomerulonephritis and retinal drusen are also associated with variants in *COL4A3* or *COL4A4*, and GBM thinning that may facilitate IgA movement into the mesangium^43^.

Individuals with macular degeneration and drusen do not develop glomerular disease^25^. This is best exemplified in dense deposit disease caused by *CFH* mutations. Despite the shared pathogenesis of glomerular immune complexes and drusen in this disease, individuals with macular degeneration do not have glomerular immune deposits, glomerulonephritis or renal impairment, in part because different CFH domains are affected by macular degeneration and dense deposit disease^11^.

Otherwise IgA glomerulonephritis has no distinctive retinal features apart from the hypertensive changes, that include vasculopathy, choroidal infarcts and serous retinal detachments^44–46^. These tend to improve with better blood pressure control.

The strengths of this study were the size of the cohort, the clinical detail, and the reproducibility of the drusen counting technique. The study’s major limitations were the insensitivity of retinal imaging for small drusen and the mainly cross-sectional nature of the study that detected drusen at a single point in time. However even if drusen numbers were underestimated using this approach the conclusions of significance remained true for drusen that were large enough to be visualised on imaging.

There are further limitations. This study did not exclude individuals with diabetes despite the possibilities of drusen being obscured by diabetic retinopathy or being confused with hard exudates. However all images were reviewed by an ophthalmologist and only two had exudates or a proliferative retinopathy. In addition, study participants were not asked about a family history of macular degeneration but this risk was minimised by the cohort age and the use of matched controls. Finally the study did not examine whether drusen counts were lower after transplantation-associated immunosuppression.

IgA glomerulonephritis is a common disease and one third of affected individuals develop kidney failure. The demonstration of drusen in IgA and other autoimmune forms of glomerulonephritis^47,48^ is consistent with complement activation in disease initiation and progression, and drusen formation represents a model for studying immune-mediated glomerular disease. Treatments are emerging that address complement activation and oxidative stress in drusen formation that may also be effective in IgA glomerulonephritis^49^.

The demonstration of drusen is a non-invasive, widely-available and inexpensive technique, and further studies may clarify their usefulness in assessing disease activity and the response to treatment in IgA glomerulonephritis.

## Data Availability

The datasets used and/or analysed during the current study are available from the corresponding author on reasonable request.
